# Indications and added value of videourodynamics in men with spinal cord injury

**DOI:** 10.1002/bco2.370

**Published:** 2024-07-08

**Authors:** Marc Françot, Chloé Lefevre, Bénédicte Reiss, Marc Lefort, Georges Karam, Jerome Rigaud, Loic Le Normand, Alain Ruffion, Brigitte Perrouin‐Verbe, Marie‐Aimee Perrouin‐Verbe

**Affiliations:** ^1^ Department of Urology Nantes University Hospital Nantes France; ^2^ Department of Physical Medicine and Rehabilitation Nantes University Hospital Nantes France; ^3^ Urology Department, Lyon Sud Hospital, Hospices Civils de Lyon, Lyon Cancer Innovation Center (EA 3738 CICLY) Lyon Sud Medical School, University of Lyon 1 Lyon France

**Keywords:** detrusor sphincter dyssynergia, endoscopic sphincterotomy, neurogenic lower urinary tract dysfunction, spinal cord injury, urodynamic study, video‐urodynamic study

## Abstract

**Purpose:**

The primary aim of this study was to evaluate the indications and additional information provided by videourodynamic study (VUDS) over urodynamic studies (UDS) in men with spinal cord injury (SCI) and neurogenic lower urinary tract dysfunction (NLUTD). The secondary aim was to determine the added value of VUDS and its impact on bladder management.

**Materials and Methods:**

Single‐centre retrospective study of all men with SCI who underwent VUDS between 2011 and 2021. Participant characteristics, clinical data and indications for UDS and VUDS as well as bladder management were recorded. The added value of VUDS was defined as additional information not provided by standard UDS that impacted on bladder management (choice of voiding mode, surgical indication or type of surgery).

**Results:**

Eighty‐eight men with a median age of 52 years were included. In 20 men who were unable to perform self‐catheterisation, the VUDS clarified the nature and extent of the obstruction and enabled targeted surgery to achieve reflex bladder emptying in all of them. VUDS also clarified the type and level of obstruction in 28 patients, enabling targeted surgery in 24. In 11 men, VUDS was performed as part of the preoperative assessment for a Brindley procedure or after this operation if a complication occurred during follow‐up to confirm the need for further surgery or to target surgical revision. Overall, VUDS had added value in 59 patients (67%).

**Conclusions:**

VUDS had added value over UDS in specific situations; the additional information provided impacted on bladder management in men with SCI and NLUTD.

## 1 INTRODUCTION

The good urodynamic practices published in 2011 by the International Continence Society (ICS)^1^ indicate that ‘invasive’ urodynamic studies (UDS) may be combined with imaging.^2^ Invasive UDS performed with a contrast agent during bladder filling is called a videourodynamic study (VUDS) and was first described in the 1970s.^3^ VUDS combines standard cystometry with retrograde urethrocystography during bladder filling and emptying. This examination allows for a functional and morphological assessment of the lower urinary tract coupled with a morphological assessment of the upper urinary tract.^4^

Although the level of evidence for the added value of VUDS is low and data in the literature data are insufficient to justify its routine use in the assessment of lower urinary tract dysfunction (LUTD), VUDS is considered the examination of choice for the assessment of neurogenic lower urinary tract dysfunction (NLUTD) and is recommended by the European Association of Urology (EAU), American Urological Association (AUA), Society of Urodynamics, Female Pelvic Medicine and Urogenital Reconstruction (SUFU) and the International Consultation on Incontinence.^2^, ^4^, ^5^, ^6^, ^7^, ^8^

Despite these recommendations, VUDS is not always performed in people with NLUTD, depending on the centre and habits of each country. This may be because VUDS requires a technical platform that is suitable for performing radiological examinations and that is accessible to people with neurological impairment. In addition, this examination requires a team trained in both urodynamics and radiation protection.^9^, ^10^

In our tertiary care neuro‐urology centre, VUDS is commonly performed to explore NLUTD, especially in men with SCI.

The primary aim of this study was to evaluate the indications for VUDS and to identify additional information provided by VUDS over UDS in men with spinal cord injury (SCI) and neurogenic lower urinary tract dysfunction (NLUTD).

The secondary aim was to determine the added value of VUDS compared to standard UDS and its impact on bladder management.

## 2 MATERIALS AND METHODS

We conducted a single‐centre, retrospective study using data from the Department of Urology and Physical Medicine and Rehabilitation and included all men with SCI who had undergone VUDS between 2011 and 2021, either during their first evaluation or during a neuro‐urological follow‐up consultation. Men under 18 years of age, with no previous standard UDS, and who were not regularly followed in our centre were excluded.

All VUDSs were performed in a lead‐lined room either in the Urology Department or in the radiology examination room in the Department of Physical Medicine and Rehabilitation. Both rooms comply with radiation protection measures (lead‐lined room, standard‐compliant fluoroscopy equipment and protective shields). VUDS was performed with the individual in the supine position, and the technique and procedure followed the ICS recommendations.^5^ Imaging was performed according to a local protocol adapted from the latest edition of *Abrams' Urodynamics* ^11^ and recent publications to limit radiation exposure to individuals and staff.^9^, ^10^ All men underwent urethrocystoscopy before VUDS to rule out anatomical obstruction.

The study protocol was declared to the French national data authority (CNIL) under reference 2227220v0.

### 2.1 Data collected

The indications for UDS and VUDS, the clinical and urodynamic data obtained from each technique and the subsequent management strategy were collected from the men's medical records. We identified additional information provided by VUDS and evaluated its impact on bladder management.

The added value of VUDS was defined as the acquisition of additional information beyond that obtained from standard UDS, which impacted bladder management.

An impact on bladder management was defined as a confirmation or change in the chosen voiding method, and/or a change or adjustment in the planned surgical procedure, and/or a contribution to the selection of the most suitable procedure for the individual.

### 2.2 Statistical analysis

Qualitative data are expressed as counts or percentages (*n*, %) and quantitative data as the median with the interquartile range (Q1‐Q3).

## 3 RESULTS

Between 2011 and 2021, 7333 urodynamic examinations were performed at our centre, including 202 VUDSs (2.7%). Eighty‐eight men with SCI were included in the analysis. The median age was 52 (42–68) years. The characteristics of the participants are shown in Table 1.

**TABLE 1:** Participants' characteristics *n* = 88.

Age (years) (median, IQR)	52 (42–68)
BMI (kg/m^2^) (median, IQR)	25 (22–28)
Neurological level of the lesion, *n* (%)	
C1–C8	68 (77%)
Of which ≤C6	53 (60%)
T1–T12	20 (23%)
AIS grade (*n*, %)	
AIS A	51 (58%)
AIS B	9 (10%)
AIS C	12 (14%)
AIS D	16 (18%)

### 3.1 VUDS performed at the initial phase of SCI

Twenty (23%) men were assessed in the initial phase of SCI when they were hospitalised in the Spinal Unit (Table 2). All had high‐level tetraplegia (>C5) were unable to self‐catheterise and were considered potential candidates for reflex bladder emptying in a penile sheath, depending on detrusor contractility.

**TABLE 2:** Characteristics of the patients assessed at initial phase (Spinal Unit).

Voiding mode	Intermittent catheterisation (by nurses) *n* = 20
Results of UDS	BOO
Indication VUDS	To precise nature and location of the obstruction
Results of VUDS	DSD in *n* = 20 including 8 with no bladder neck opening
Treatment	ES *n* = 20 (including 8 incision of the bladder neck)
Added value of VUDS	*N* = 20

Abbreviations: BOO, bladder outlet obstruction; DSD, detrusor sphincter dyssynergia; ES, endoscopic sphincterotomy. UDS, urodynamics; VUDS, videourodynamics.

**FIGURE 1:**
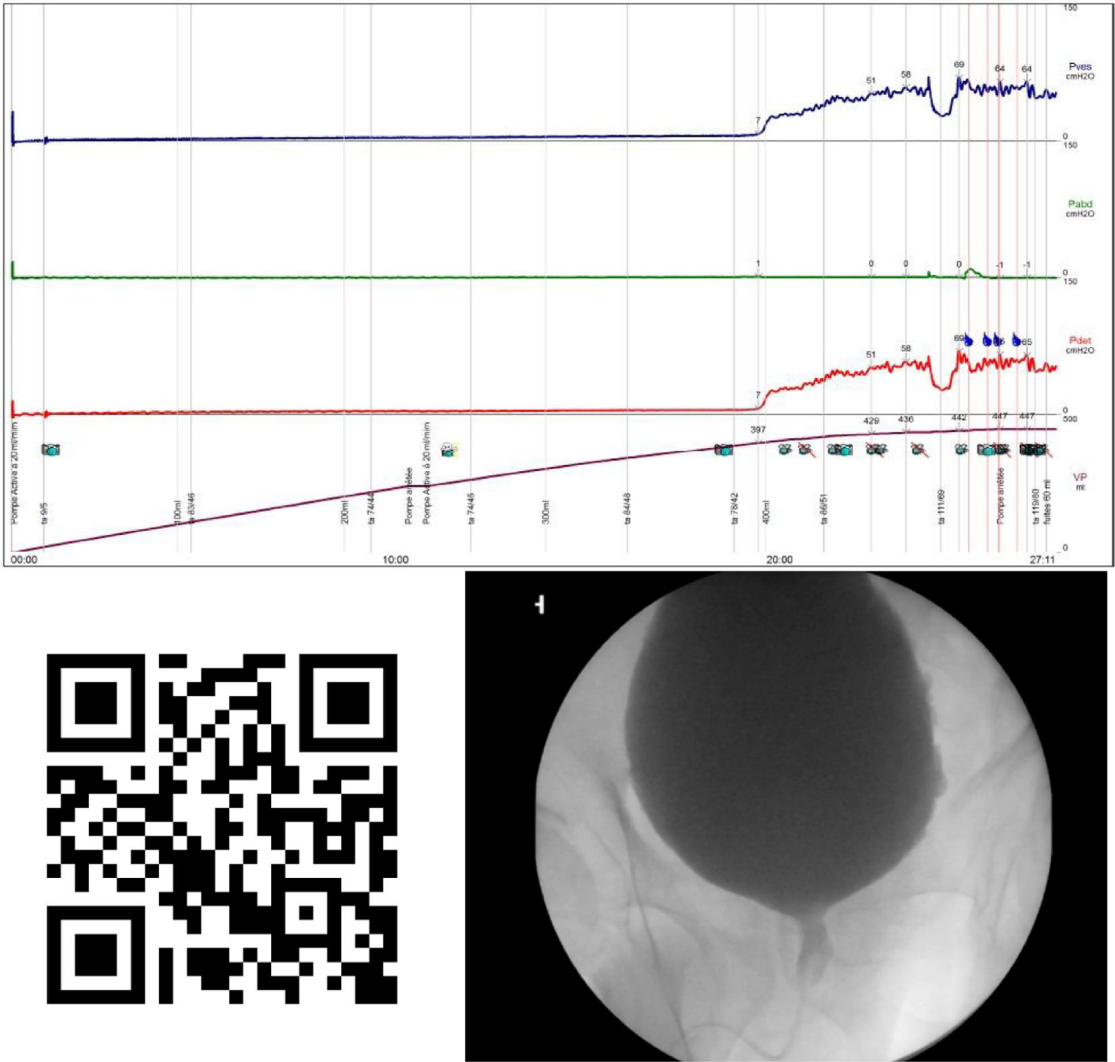
VUDS confirming detrusor sphincter dyssynergia. UDS traces (2A): BOO with prolonged detrusor contraction, with high amplitude. At the end of contraction, leakage is observed during intermittent opening of the striated sphincter. In video (2B), a wide opening of the bladder neck with dilatation of the proximal urethra over a narrow striated sphincter is observed during detrusor contraction, confirming an isolated striated DSD, without detrusor bladder neck dyssynergia. At the end of the contraction, there is an intermittent opening of the striated sphincter with leakage of contrast into the urethra (link to video).

All these men initially underwent clean intermittent catheterisation performed by nurses. All these men were found to have bladder outlet obstruction (BOO) on UDS, with sufficient detrusor contractility to consider reflex bladder emptying.^12^, ^13^, ^14^ VUDS was performed to determine the nature and location of the BOO before surgery. VUDS confirmed the presence of detrusor sphincter dyssynergia (DSD) (functional obstruction of the striated sphincter) (Figure 1) in all 20 men and a lack of concomitant bladder neck opening in eight men. All men underwent endoscopic sphincterotomy (ES), and eight had a simultaneous incision of the bladder neck. VUDS had an added value in all 20 (100%) men.

### 3.2 VUDS performed during follow‐up

Sixty‐eight (77%) men underwent VUDS during their neuro‐urological follow‐up.

In 44/68 men, the voiding mode was reflex bladder emptying (Table 3), including 32/44 (72.7%) men with a history of ES. UDS was performed during follow‐up for complications (Table 3).

**TABLE 3:** Characteristics of the patients during follow up (reflex bladder emptying).

Voiding mode	Reflex bladder emptying follow up *n* = 44 32 history of ES				
Indication UDS	Increased PVR +/− AD symptomatic +/− UTI *n* = 44				
Results UDS	BOO				Detrusor hypocontractility *n* = 22
Indication VUDS	Specify the nature and location of BOO				Verify the absence of concomitant bladder outlet abnormality
Results	Confirmation of DSD *n* = 16	No bladder neck opening *n* = 1	Urethral stricture *n* = 2	Prostatic obstruction *n* = 3	No bladder outlet abnormality *n* = 22
Treatment	ES *n* = 13 (Second ES for 6)	Isolated incision of the bladder neck *n* = 1	Urethrotomy n = 2	TURP *n* = 3	Non continent diversion, *n* = 6 Monitoring *n* = 16
Value‐added VUDS	*N* = 13	*N* = 1	*N* = 2	*N* = 3	*N* = 0

Abbreviations: AD, autonomic dysreflexia; DSD, detrusor sphincter dyssynergia; ES, endoscopic sphincterotomy; PVR, post‐void residual; TURP, transurethral resection of the prostate; UDS, urodynamics; UTI, urinary tract infection; VUDS, videourodynamics.

In 22/44 men (50%), UDS showed the presence or recurrence of BOO. VUDS was performed to determine the nature and location of the BOO. In 16 men, VUDS confirmed DSD, allowing isolated ES to be performed in 13 men (including six who had previously undergone ES). The remaining three men did not undergo surgery because they had complete bladder emptying without complications at the next assessment. In six men, VUDS revealed another site of BOO that led to targeted surgery (details in Table 3). VUDS had an added value for 19 men.

In the remaining 22/44 men (Table 3), VUDS confirmed secondary detrusor underactivity (maximum detrusor pressure during reflex detrusor contraction <40 cmH2O). Of these, 16 underwent monitoring (complete bladder emptying without autonomic dysreflexia or urinary tract infection). The remaining six men underwent non‐continent urinary diversion because of high post‐void residual and complications. VUDS provided no additional information and had no impact on the management of these 22 men (details in Table 3).

Overall, during the follow‐up of men with reflex bladder emptying, VUDS had an added value for 19/44 (43%) men (Table 3).

In 9/68 men with incomplete SCI (AIS D), the voiding mode was spontaneous voiding (Table 4). UDS was performed for both underactive bladder (slow urine stream, hesitancy and straining to void) and overactive bladder (OAB) syndromes. The purpose of VUDS was to specify the nature and location of the BOO found by UDS. VUDS confirmed five cases of DSD. Intermittent self‐catheterisation with anti‐muscarinic drugs was proposed to all five men as they were all able to self‐catheterise (gold standard treatment). In the four remaining men, VUDS revealed the absence of DSD, and the obstruction was located either at the level of the bladder neck (*n* = 2) or at the prostate (*n* = 2); this information allowed targeted surgical treatment (details in Table 4) and continuation of spontaneous voiding with complete bladder emptying. VUDS had an added value for all nine (100%) men.

**TABLE 4:** Characteristics of the patients during follow‐up (spontaneous voiding or intermittent self‐catheterisation).

Voiding mode	Spontaneous voiding (AIS D) *n* = 9			Self‐catheterisation *n* = 4
Indication UDS	UAB *n* = 6 OAB syndrome *n* = 3			Failure of anti‐muscarinic drugs *n* = 3 Failure of botulinum toxin A *n* = 1
Results UDS	BOO *n* = 9			BOO *n* = 4
Indication VUDS	Specify the nature and location of the obstruction			Specify the nature and location of the obstruction
Results	Isolated DSD *n* = 5	No bladder neck opening *n* = 2	Prostatic obstruction *n* = 2	BDS *n* = 4
Treatment	Intermittent self‐catheterisation + anti‐muscarinic drugs *n* = 5	Isolated incision of the bladder neck *n* = 2	TURP *n* = 2	Self‐catheterisation + botulinum toxin injections *n* = 3 Self‐catheterisation + enterocystoplasty *n* = 1
Value‐added VUDS	*N* = 5	*N* = 2	*N* = 2	*N* = 0

Abbreviations: DSD, detrusor sphincter dyssynergia; ES, endoscopic Sphincterotomy; OAB, overactive bladder; TURP, transurethral resection of the prostate; UAB, underactive bladder; UDS, urodynamics; VUDS, videourodynamics.

Four other men were already performing intermittent self‐catheterisation at the time of VUDS (Table 4). All of them had refractory neurogenic detrusor overactivity (NDO) found on UDS (three anti‐muscarinic drug failures and one botulinum toxin A failure). VUDS confirmed isolated DSD in all four men. VUDS had no added value for these four men, as they were still able to self‐catheterise and as intermittent self‐catheterisation is the preferred option in such cases, rather than reflex bladder emptying after sphincterotomy. For these four men, VUDS did not provide additional information that could impact bladder management. The refractory NDO was treated by adjusting their therapy.

### 3.3 VUDS performed for initial selection or follow‐up after SARS surgery

In seven men (Table 5), VUDS was performed as part of the preoperative assessment for a Brindley procedure (sacral deafferentation and sacral anterior sacral roots stimulation: SDAF/SARS). All men performed self‐catheterisation and had complete spinal cord injuries (AIS A). The Brindley procedure has been proposed as an alternative to self‐catheterisation.^15^ All men had BOO at initial assessment, suggestive of DSD, found on UDS. VUDS confirmed DSD in all seven men with bladder neck incompetence and in two men who did not undergo surgery because of a high risk of de novo stress urinary incontinence.^16^

**TABLE 5:** Initial selection for Brindley procedure or follow‐up after Brindley.

Voiding mode	Self‐catheterisation *n* = 7	Brindley follow‐up *n* = 4
Indication UDS	Assessment before Brindley surgery	Increasing PVR or AD
Results UDS	BOO	Urodynamic obstructive syndrome
Indication VUDS	Specify the nature and location of obstruction Assessment of bladder neck competence during filling	Specify the nature and location of obstruction
Results	DSD *n* = 7 including 2 with bladder neck incompetence	DSD *n* = 2 No opening of the bladder neck *n* = 2
Treatment	Brindley *n* = 5 Self‐catheterisation + botulinum toxin *n* = 2	Second ES *n* = 2 Bladder neck incision *n* = 2
Value‐added VUDS	*N* = 7	*N* = 4

Abbreviations: AD, autonomic dysreflexia; DSD, detrusor sphincter dyssynergia; ES, endoscopic sphincterotomy; OAB, overactive bladder; PVR, post‐void residual; SARS, sacral anterior root neurostimulation; UDS, urodynamics; VUDS, videourodynamics.

In four other men, UDS was performed after the Brindley procedure (Table 5) because of increased post‐void residual with complications or recurrence of autonomic dysreflexia and/or recurrence of conus medullaris reflex found at the clinical examination. UDS found a recurrence of BOO during electro‐induced micturition. In VUDS, recurrence of DSD was found in two men, and secondary non‐opening of the bladder neck was found in another two men, which allowed appropriate surgical treatment to be planned (details in Table 5).

VUDS had an added value in all 11 men (100%) who had a planned Brindley procedure or during follow‐up after a Brindley procedure.

Overall, VUDS added value over standard UDS in 59 (67%) men with SCI.

## 4 DISCUSSION

In this study, we aimed to investigate the specific indications for VUDS in our centre and its added value for bladder management in men with SCI. An added value of VUDS was found in 67% of cases.

To our knowledge, this is the first study to provide evidence for the added value of VUDS for the management of NLUTD in men with SCI, supporting current recommendations.

VUDS is routinely performed at our centre in men with SCI, either to guide the initial bladder management and choice of voiding mode or during follow‐up. Our treatment algorithm is in line with the recommendations for the treatment of NLUTD.^5^, ^15^

Around 70%–80% of people with SCI have NDO with or without DSD. NDO is associated with a risk of upper urinary tract impairment if not treated appropriately.^17^ The gold standard of care is intermittent self‐catheterisation associated with treatment for the NDO.^15^, ^18^

In men who are unable to self‐catheterise, reflex bladder emptying after ES should be considered if there is sufficient reflex detrusor contractility. This solution requires the use of a penile sheath to collect urine.^13^, ^15^, ^19^ An alternative to this is the Brindley procedure, but it is only suitable for individuals with complete lesions (AIS A) and no bladder neck incompetence.^15^, ^16^ In a few men with incomplete SCI (AIS D) and with balanced bladder emptying, spontaneous voiding may be proposed.

The main reasons for performing VUDS in this case series were either for the initial assessment, before considering reflex bladder emptying (men with high‐level tetraplegia unable to perform self‐catheterisation) or as part of the follow‐up for those who experienced complications with reflex voiding.

VUDS was only performed in men with BOO (found using UDS) and sufficient detrusor contractility to consider reflex bladder emptying. VUDS confirmed DSD, characterised by involuntary contraction of the striated sphincter during detrusor contraction with dilatation of the proximal urethra (Figure 1).^20^, ^21^, ^22^ In some cases, other levels of obstruction were also identified, such as a lack of opening of the bladder neck (detrusor bladder neck dyssynergia [DBND]). These findings may influence the choice of voiding mode and type of surgery, thus demonstrating the added value of VUDS.

In some series of ES, concomitant bladder neck incision was systematically performed without any prior videourodynamic evaluation.^23^ However, a recent study by Wang et al.,^22^ involving 647 men with SCI who underwent VUDS to assess the type of NLUTD and the UDS pattern, found DBND in less than 10% of participants.

Systematic concomitant bladder neck incision during ES therefore does not appear to be necessary in 90% of men. It may also increase per‐ and postoperative bleeding, resulting in permanent stress urinary incontinence and retrograde ejaculation during sexual intercourse.^12^, ^19^, ^23^

An alternative to VUDS for the diagnosis of DSD is retrograde cystography. However, the morphological assessment is not synchronised with bladder pressure monitoring during UDS.^11^, ^17^ Therefore, findings may indicate an absence of micturition or an absence of detrusor contraction during the examination or true DBND (lack of bladder neck opening during detrusor contraction). Another limitation of retrograde cystography is the lack of training of radiological staff in handling people with neurological disorders, particularly those with SCI (autonomic dysreflexia during bladder filling, reactivation of reflex contraction).

Performing electromyography (EMG) in conjunction with cystometry is an alternative method for the diagnosis of DSD.^11^, ^17^ However, this method may not detect other types or locations of obstructions, such as DBND. Therefore, in such cases, VUDS is the assessment of choice, especially when assessing DSD in people with SCI, as a morphological assessment is necessary for decision‐making.^24^ In addition, EMG does not always detect DSD, and VUDS may be the only effective detection method.^21^

Finally, in our series, VUDS had an added value in men with spontaneous voiding found to have BOO on UDS as it identified the nature and location of the obstruction.

**FIGURE 2:**
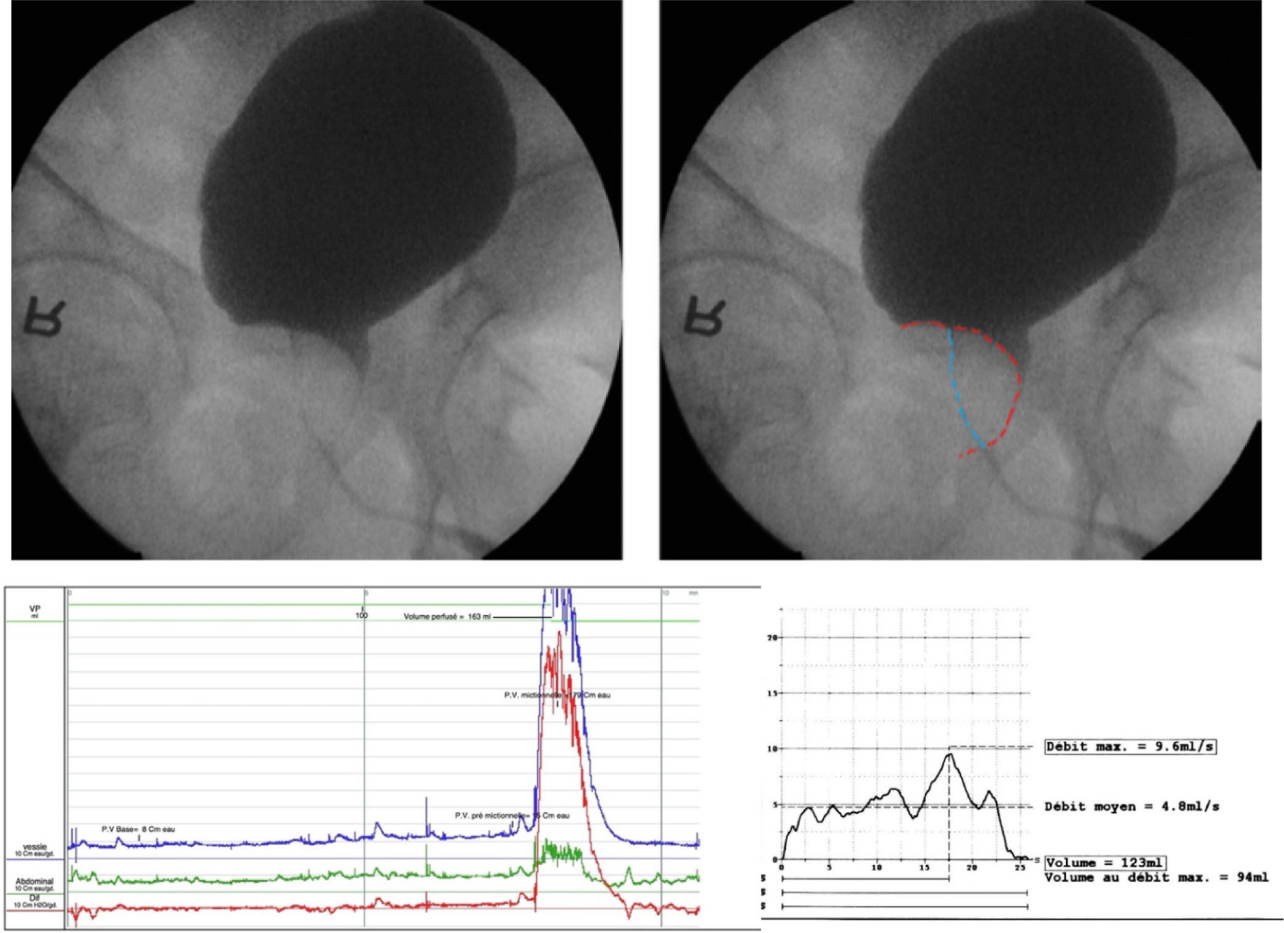
Video urodynamic examination confirming BOO at the level of the prostate. On UDS traces, BOO is observed (high detrusor pressure during voiding, associated with a low flow). On the video, the outline of the prostatic gland is marked in red and the course of the prostatic urethra in blue. *Source*: Pictures from *Abrams' Urodynamics 4th Edition*.

In summary, in cases of BOO with sufficient detrusor contractility, for which reflex bladder emptying or spontaneous voiding were indicated, VUDS had a real added value. The additional information provided by VUDS, particularly the identification of the type and location of obstruction, had an impact on bladder management (Figure 2).

It should be noted that for non‐neurological disorders, VUDS is recommended for young people with BOO, especially when primary bladder neck obstruction is suspected.^8^, ^25^

This work also identified situations in which VUDS did not provide an added value over UDS (35% of cases). These situations involved secondary detrusor underactivity in men with reflex bladder emptying or with NDO+/−DSD who could self‐catheterise. In these cases, identifying the type and location of the obstruction (anatomical or functional) is not of interest, in our opinion, as it would probably not have been treated.^13^, ^19^

VUDS may also be useful to detect reflux towards the upper urinary tract and to determine if it is associated with high endovesical pressure.^6^ We did not find reflux in our cohort, probably because our study sample underwent close neuro‐urological monitoring, which allowed the maintenance of a low‐pressure bladder.

Finally, VUDS also has an impact on treatment in very specific situations, such as before a Brindley procedure or as part of the follow‐up after this procedure. Before surgery, VUDS was used to verify the competence of the bladder neck in 5/7 individuals with thoracolumbar lesions and confirmed the indication for surgery. Indeed, bladder neck incompetence may be found in people with thoracolumbar lesions and can increase the risk of de novo stress urinary incontinence after a Brindley procedure.^16^

VUDS also helped to identify the type and location of BOO during follow‐up, which was then treated with targeted surgery.

**FIGURE 3:**
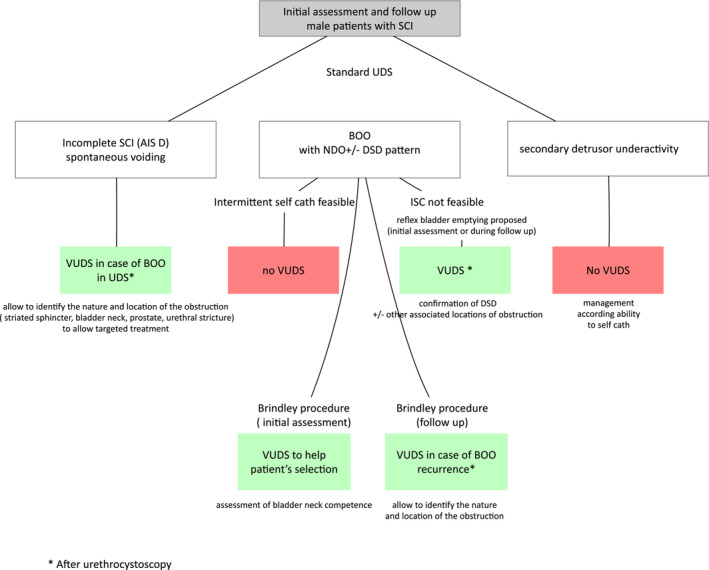
Indications for VUDS in the bladder management of male patients with SCI.

Based on these results and our experience, we propose an algorithm to specify the indications for VUDS as part of the bladder management of men with SCI (Figure 3).

One of the limitations of our study is its retrospective and single‐centre design and recruitment bias. Indeed, the sample consisted only of people with SCI with mainly complete cervical spine injuries who have very specific indications for VUDS.

## 5 CONCLUSION

VUDS was useful for the evaluation of NLUTD in men with SCI. In specific situations, it added value over standard UDS; the additional information provided impacted bladder management, whether performed before surgery or at follow‐up, and allowed for more targeted treatment.

## AUTHOR CONTRIBUTIONS

Not applicable.

## CONFLICT OF INTEREST STATEMENT

The authors declare no conflicts of interest.
